# Occlusal Adjustment in Free‐End Implant‐Supported Restorations: Digital Scans Versus Conventional Impressions With Full‐ and Half‐Arch Techniques

**DOI:** 10.1002/cre2.70454

**Published:** 2026-09-17

**Authors:** Mohammadreza Nakhaei, Hossein Dashti, Arsalan Shahri, Mahsa Rahmani, Ali Kazemian, Saeid Sabzevari, Masih Rezaee

**Affiliations:** ^1^ Department of Prosthodontics, School of Dentistry Mashhad University of Medical Sciences Mashhad Iran; ^2^ Dental Materials Research Center, Mashhad Dental School Mashhad University of Medical Sciences Mashhad Iran; ^3^ Department of Prosthodontics, School of Dentistry Bojnurd University of Medical Sciences Bojnord Iran; ^4^ Department of Community Oral Health, School of Dentistry Mashhad University of Medical Sciences Mashhad Iran

**Keywords:** CAD‐CAM, dental impression techniques, digital impression, intraoral scanners, occlusal adjustment

## Abstract

**Objectives:**

To quantify the occlusal adjustment required for two‐unit, implant‐supported polymethyl‐methacrylate (PMMA) crowns with either digital or conventional impression techniques, using full‐arch and half‐arch workflows.

**Material and Methods:**

An acrylic maxillary–mandibular model carrying two bone‐level implant analogs (sites 46–47) was mounted on a semi‐adjustable articulator. Twenty impressions were made (*n* = 5 per group): digital full‐arch, digital half‐arch, conventional full‐arch, and conventional half‐arch. PMMA crowns were designed using computer‐aided design (CAD) software, milled, and surface‐scanned before adjustment. Occlusal contacts were refined on the articulator with 21 µm film while recording adjustment time. Pre‐ and post‐adjustment scans were superimposed and analyzed using three‐dimensional surface analysis software to quantify mean material reduction (µm). Data were analyzed with one‐way ANOVA and independent‐samples *t*‐tests (*α* = 0.05).

**Results:**

Digital workflows required significantly less occlusal reduction (146 ± 35 µm vs. 226 ± 35 µm; *p* = 0.035) and shorter adjustment times (144 ± 36 s vs. 385 ± 36 s; *p* < 0.001) than conventional, with no effect of arch span (full vs. half; *p* > 0.05). Within protocols, the digital half‐arch group showed the smallest adjustment (127 ± 37 µm) and fastest finishing (98 ± 33 s), whereas the conventional half‐arch group required the longest time (456 ± 21 s).

**Conclusion:**

Fully digital impressions reduce both the amount of occlusal adjustment and the chairside time needed to seat implant‐supported crowns compared with conventional silicone impressions. Reducing the scan to a half‐arch does not compromise occlusal precision, making the digital half‐arch workflow an efficient alternative for posterior, free‐end implant restorations.

**Clinical Relevance:**

In implant posterior PMMA crowns, the impact of impression/workflow choice on occlusal adjustment is unclear. This in‐vitro study measured occlusal reduction and chairside time across digital versus conventional and full‐ versus half‐arch workflows. Digital, especially half‐arch, required less adjustment and shorter time. Clinically, a digital half‐arch approach may streamline delivery and reduce unnecessary material removal, pending confirmation in patient‐based settings.

## 1 Introduction

Dental implants are currently regarded as one of the most preferred treatment options for replacing missing teeth because they look completely natural, are indistinguishable from the patient's own dentition, and allow full comfort during speaking and chewing (Lin et al. 2015). Nevertheless, implant‐supported therapy generally entails higher costs and longer treatment times than conventional alternatives such as tooth‐supported fixed prostheses. One way to achieve a more favorable cost‐to‐benefit ratio while still delivering a high‐quality definitive prosthesis is to adopt new technologies that shorten the overall clinical and laboratory workflow (Joda and Brägger 2016).

The impression step initiates fabrication of implant‐supported prostheses and influences every subsequent stage and long‐term success (Flügge et al. 2018), and its precision is critical, as any prosthesis‐implant misfit generates internal stresses (De La Cruz et al. 2002). Conventional impressions, despite the accuracy gains of additive silicones, remain uncomfortable for patients, whereas digital impressions bypass tray selection, material setting, disinfection, and laboratory transport, substantially improving comfort (Patzelt et al. 2014; Ahmed et al. 2024; Rojas‐Rueda et al. 2025). Since CAD‐CAM entered dentistry in 1980, its three‐step workflow—data acquisition, computer‐aided design, and computer‐aided manufacturing—has evolved rapidly. By 2003, it enabled scanning, 3‐D modeling, and fabrication of single‐unit restorations (Mizumoto and Yilmaz 2018). Since 2004, coded healing abutments have supplied precise 3‐D data on implant position relative to adjacent structures (Mizumoto and Yilmaz 2018), as STL files that feed directly into CAD‐CAM to mill models, customize abutments, and fabricate superstructures (Joda et al. 2000). To obtain restorations with acceptable morphology while minimizing chairside adjustments, it is essential to transfer the occlusal relationship accurately into the software (Queiroz et al. 2021). Another benefit of digital impressions is that, because no intra‐oral bite‐registration material is used, the likelihood of mandibular deviation from centric relation during record taking is reduced (Ender et al. 2016).

Occlusion and occlusal contacts are critical topics in dentistry (Reich et al. 2010; Gözen and Güntekin 2025); understanding their quality and distribution is necessary because the pattern of occlusal loading and its effect on biological structures influence diagnosis and treatment planning (Makino et al. 2013; Muric et al. 2019; Aldowish et al. 2024). Numerous methods have been proposed for assessing occlusion (Sigvardsson et al. 2021). Today, occlusal indicators are used to visualize contacts (Makino et al. 2013; Sigvardsson et al. 2021) and are classified as qualitative or quantitative. Qualitative tools—such as articulating paper, foils, and waxes—show the number and location of contacts, whereas quantitative systems such as T‐Scan and Occlusion‐Photo measure the intensity and duration of contacts (Makino et al. 2013). A common issue in routine prosthodontics is the discrepancy between the occlusion of a restoration on the cast and in the mouth; during the try‐in stage, this can lead to extensive occlusal grinding and loss of morphology and established contacts (Makino et al. 2013). One of the best ways to adjust a crown is therefore to make the adjustments on the cast and the provisional prosthesis (Ren et al. 2021), allowing the clinician to evaluate occlusal morphology, function, contour, and esthetics before fabricating the definitive prosthesis, saving both time and cost (Di Fiore et al. 2021).

Despite the many advantages of digital impressions, the high cost of intra‐oral scanners and the training required for new laboratory workflows are limiting factors (Papaspyridakos et al. 2016), and conventional impressions therefore remain the most widely used methods in dentistry. Because no previous study has evaluated the occlusal adjustment required for multi‐unit fixed partial dentures, and given the importance of such adjustment for occlusal morphology and contacts, the present study aimed to compare the amount of occlusal adjustment and its impact on occlusal surface morphology in implant crowns fabricated with conventional and digital impression techniques, using both half‐arch and full‐arch approaches.

## 2 Materials and Methods

### 2.1 Study Design and Setting

This in vitro laboratory study was conducted at Mashhad Dental School and was approved by the local Ethical Committee of Mashhad University of Medical Sciences with the protocol number of IR.MUMS.DENTISTRY.REC.1401.23, and is in adherence to the Declaration of Helsinki. Based on a pilot test, a two‐sample *t*‐test (*α* = 0.05, power = 0.8) indicated that five specimens per group were required.

**Figure 1:**
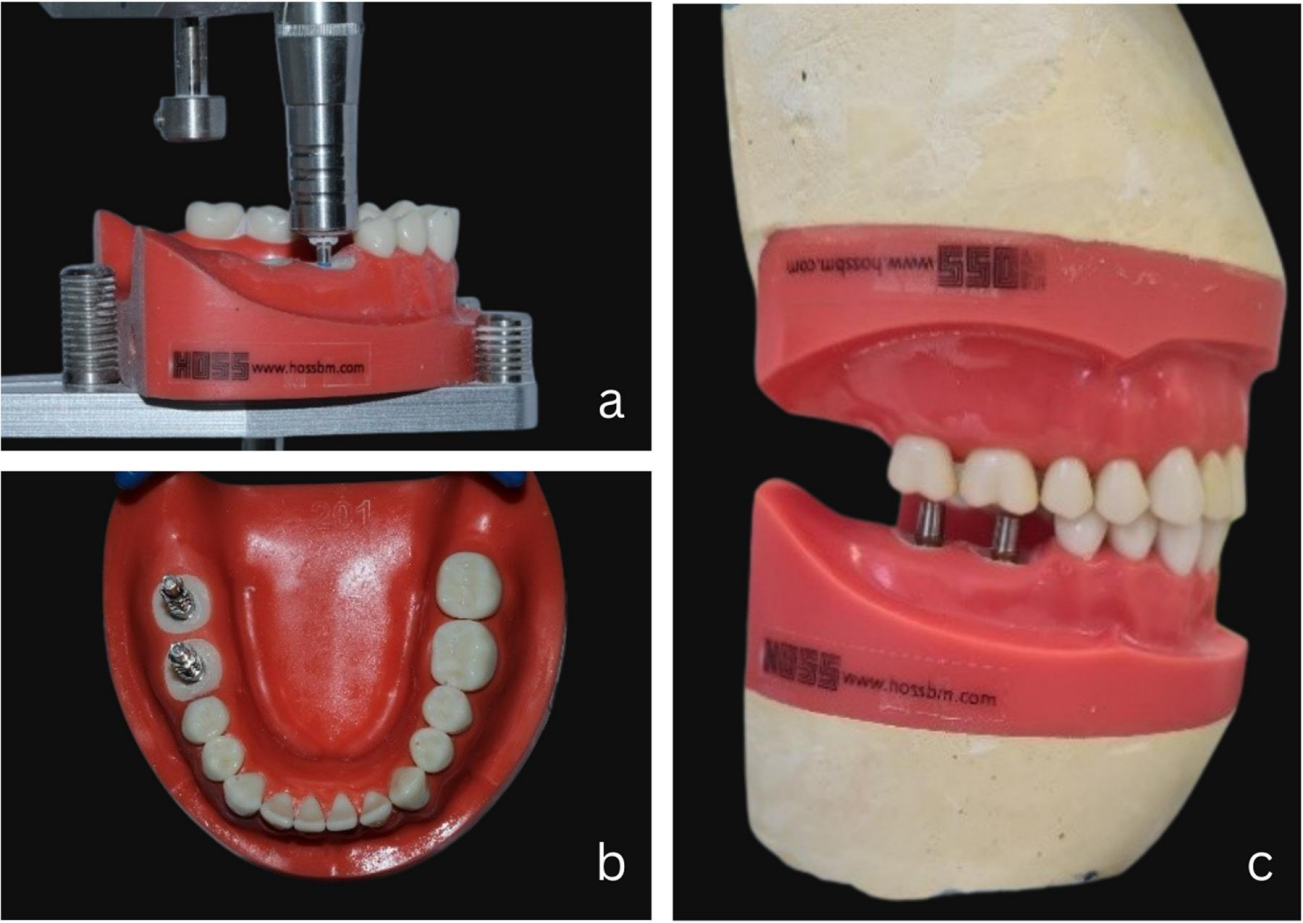
(a, b) Placement of two straight bone‐level implant analogs in the mandibular acrylic model; (c) Models mounted in articulator with baseline occlusal contacts equalized.

Mandibular first and second molars of an acrylic model were removed. Two straight bone‐level implant analogs (Dentis Fixture Lab Analogue, Dentis Inc., Daegu, Korea) were positioned in sites 46 and 47 using a surveyor and autopolymerising acrylic resin (Figure 1a,b). The acrylic maxilla‐mandible reference model was mounted on a semi‐adjustable articulator (Artex, Amann Girrbach, Austria). Baseline occlusal contacts were equalized with 21 µm articulating film (AccuFilm Fast Check, Parkell, USA; Figure 1c).

### 2.2 Impression Groups

Each impression protocol was performed five times, yielding five specimens in every group.

**Figure 2:**
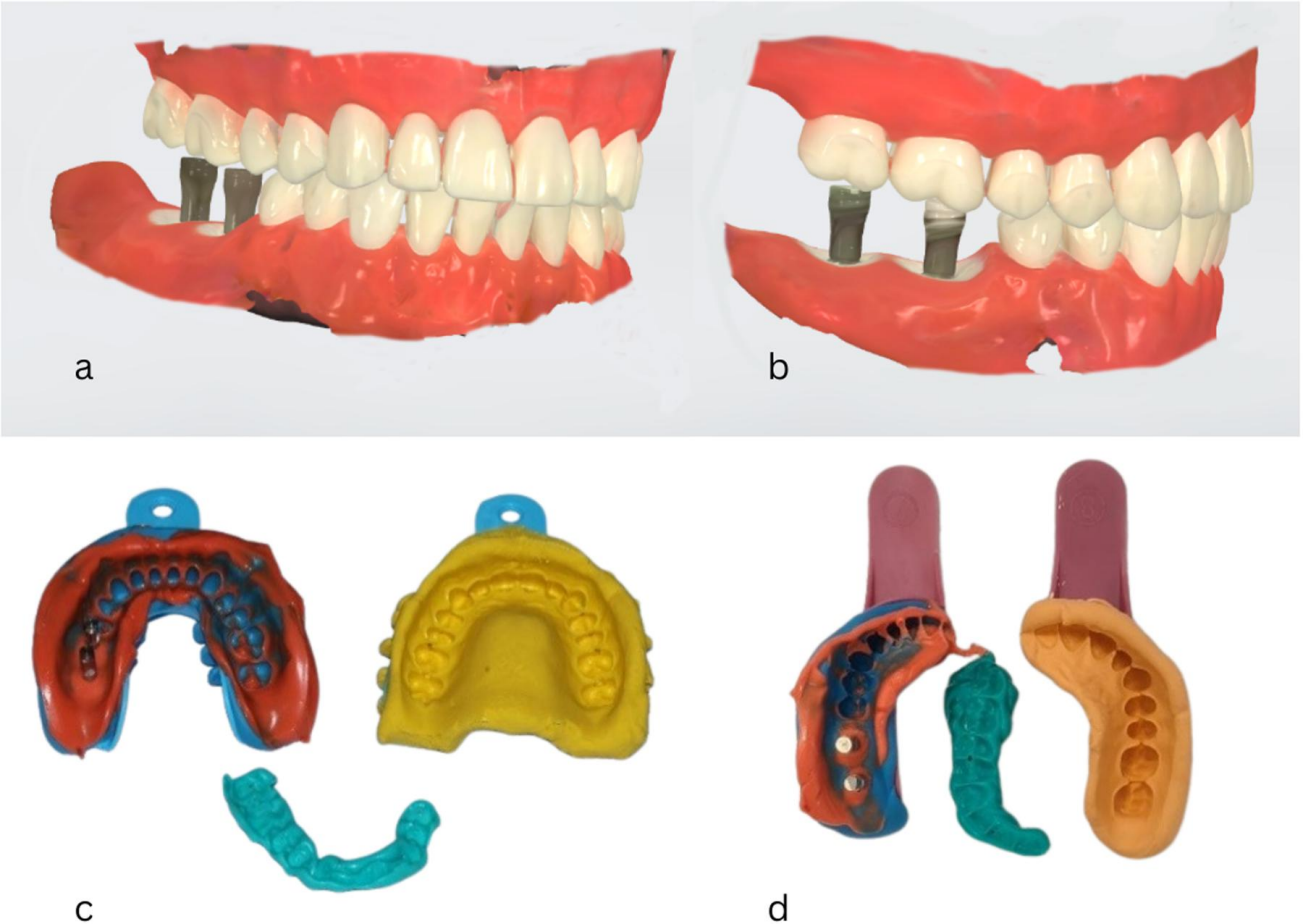
Impression protocols: (a) digital full‐arch scan with PEEK scan body and bilateral buccal registration; (b) digital half‐arch scan with unilateral buccal registration; (c) conventional full‐arch open‐tray silicone impression with pick‐up copings; (d) conventional half‐arch sectional‐tray silicone impression with abutments in place.

A. Digital, full‐arch (DF): Intra‐oral scanner (TRIOS, 3Shape, Denmark) with PEEK scan body (Arum Scan Body, CHIRIMEN, China); bilateral buccal bite‐registration (TRIOS) (Figure 2a).
B. Digital, half‐arch (DH): Same scanner and scan body; unilateral buccal bite‐registration (Figure 2b).
C. Conventional, full‐arch (CF): Prefabricated open tray; implant pick‐up copings connected, putty/light‐body addition‐silicone (Harmony Putty Soft & Harmony Light Fast Set, Elsodent, France). Bite registration with addition‐silicone material (Granit D45, Müller Omicron Dental, Germany); opposing‐arch alginate. Casts poured in type IV stone (Elite Rock Type 4 Die Dental Stone, Zhermack, Italy) and mounted on the articulator (Figure 2c).
D. Conventional, half‐arch (CH): Sectional‐tray impression as above; after impression, two straight Dentis abutments (Dentis Bone‐Level Abutment GH 1 L 5.5, Dentis Inc., Daegu, Korea) were screwed in, and a half‐arch silicone bite record was taken; opposing‐arch alginate; casts poured as in group C (Figure 2d).

### 2.3 CAD‐CAM Workflow

**Figure 3:**
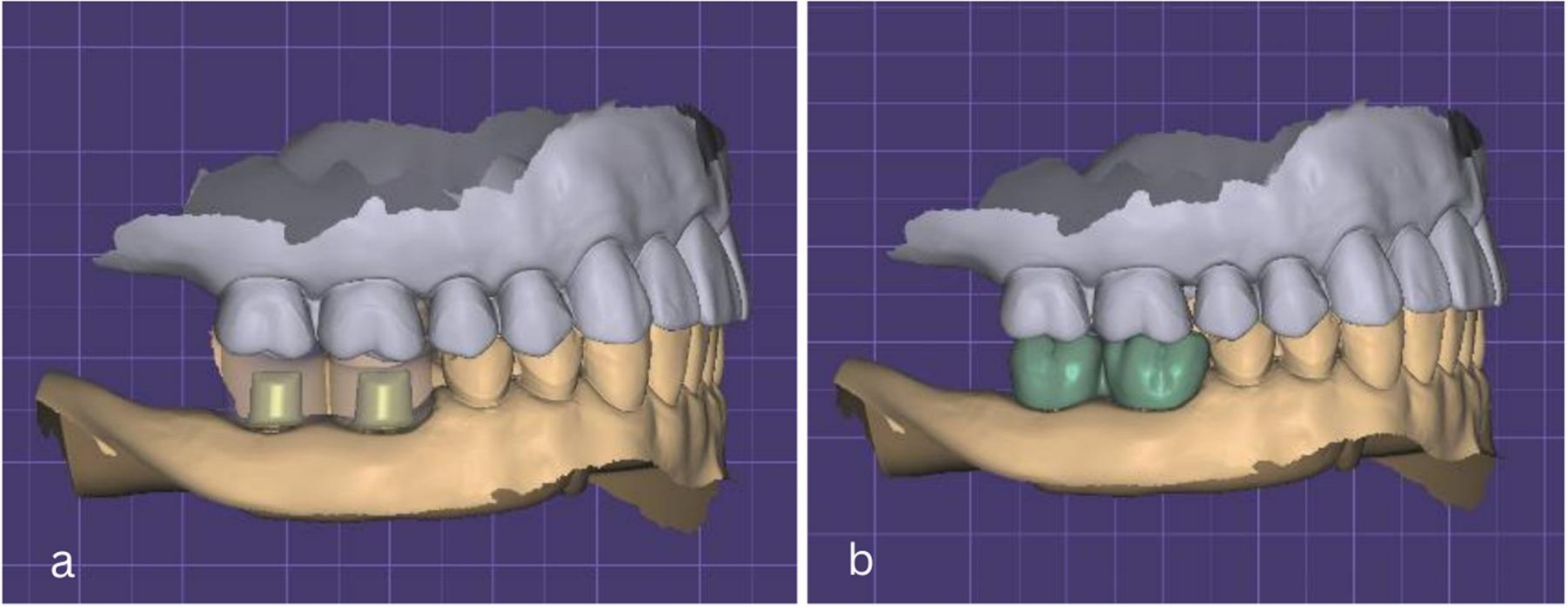
CAD‐CAM workflow for digital groups: (a) design of customized titanium abutments in exocad; (b) design of splinted full‐contour PMMA restorations in exocad.

*Digital groups:* STL files were exported to the laboratory. For each specimen, two customized titanium abutments (Customized Abutment, Dentis‐Redon, Korea) and a splinted full‐contour PMMA restoration were designed in exocad 2018 (Figure 3a,b), then milled from PMMA discs (BIL KIM, Turkey) on a five‐axis mill (vhf cam4‐S2, VHF, Germany).

**Figure 4:**
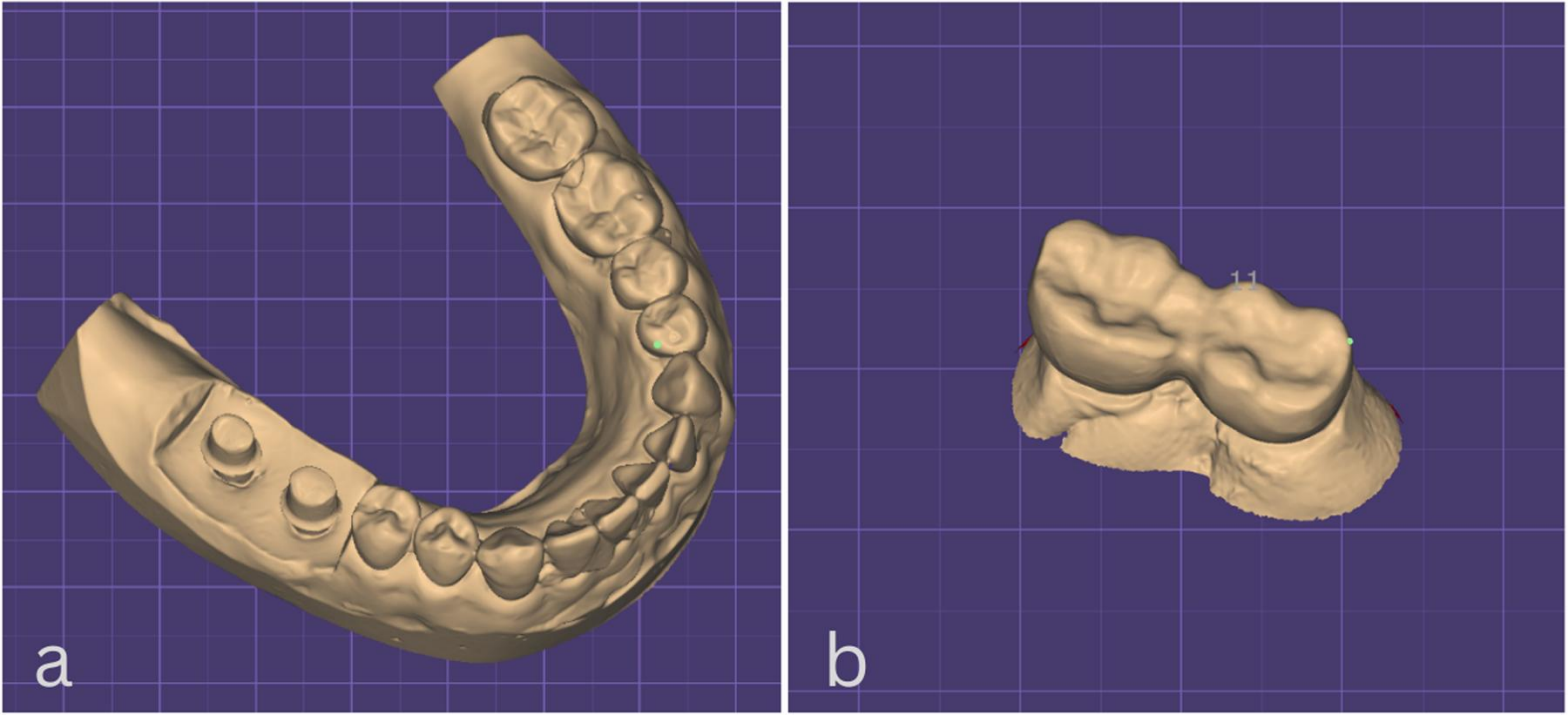
(a) Scan of customized abutments fitted on mounted casts for conventional groups; (b) laboratory scan of seated PMMA crowns to obtain pre‐adjustment STL dataset.

*Conventional groups:* Casts were mounted on the articulator; the same customized abutments were fitted and scanned (Shining 3D, China). STL files were processed in exocad 2018 (Figure 4a), and PMMA prostheses were milled identically to the digital groups.

### 2.4 Crown Placement and Pre‐Adjustment Scan

Abutments were hand‐tightened; then PMMA splinted crowns were seated, proximal contacts adjusted if required, and fit verified with a probe. Each assembly was scanned with the laboratory scanner to obtain a pre‐adjustment STL dataset (Figure 4b).

### 2.5 Occlusal Adjustment

Contacts were marked with 21 µm articulating paper (AccuFilm Fast Check, Parkell, USA) and adjusted with a straight handpiece until contacts on teeth adjacent to the implants replicated baseline. Chairside adjustment time was recorded for every sample.

### 2.6 Post‐Adjustment Scan and 3‐D Analysis

**Figure 5:**
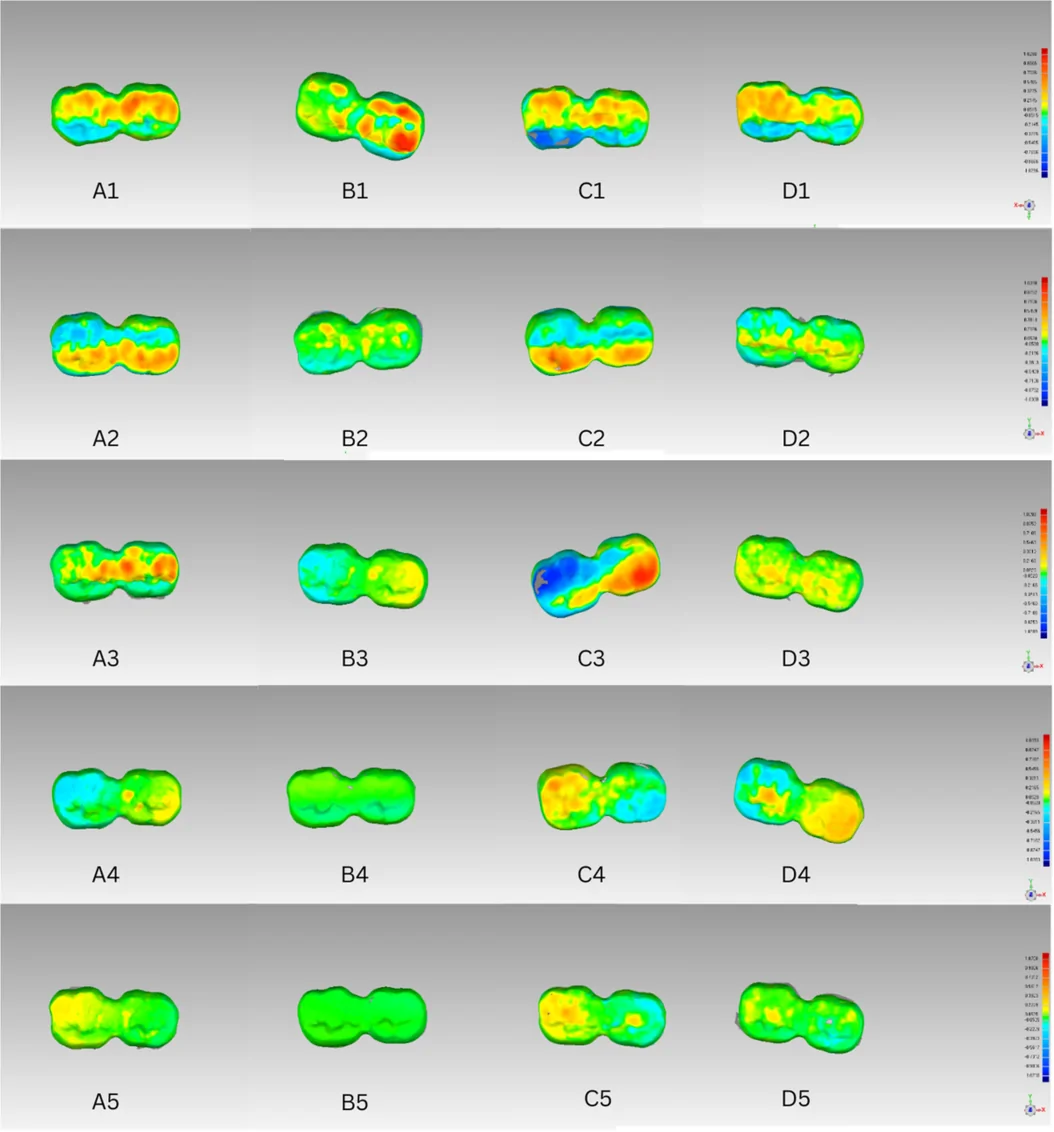
Superimposition of the before‐and‐after occlusal adjustments of all specimens (1, 2, 3, 4, and 5) of groups (A, B, C, D) in Geomagic 2021 software.

After adjustment, a second scan was taken. Pre‐ and post‐adjustment STL files were imported into Geomagic Wrap 2021 (3D Systems, USA); crowns were trimmed at the gingival margin, aligned with a best‐fit algorithm, and mean spatial discrepancy (µm) calculated to quantify material removed (Figure 5). Occlusal contact areas and morphological changes were visually compared.

### 2.7 Statistical Analysis

With limited expected variability, five repetitions per group were sufficient. Data normality was assessed descriptively; normally distributed data were compared with independent‐samples *t*‐tests (digital vs conventional; full‐arch vs half‐arch). Non‐normal data were analyzed with the Mann–Whitney *U* test. Significance was set at *α* = 0.05.

## 3 Results

Twenty specimens in four groups (*n* = 5) went under assessment. Shapiro–Wilk tests confirmed normal distribution for both outcome variables (occlusal‐adjustment amount and adjustment time) in every group (*p* > 0.05).

### 3.1 Comparison Among the Four Impression Protocols

One‐way ANOVA demonstrated significant between‐group differences in both adjustment amount (*p* = 0.034) and adjustment time (*p* < 0.001) among the four DF, DH, CF, CH protocols.

**Figure 6:**
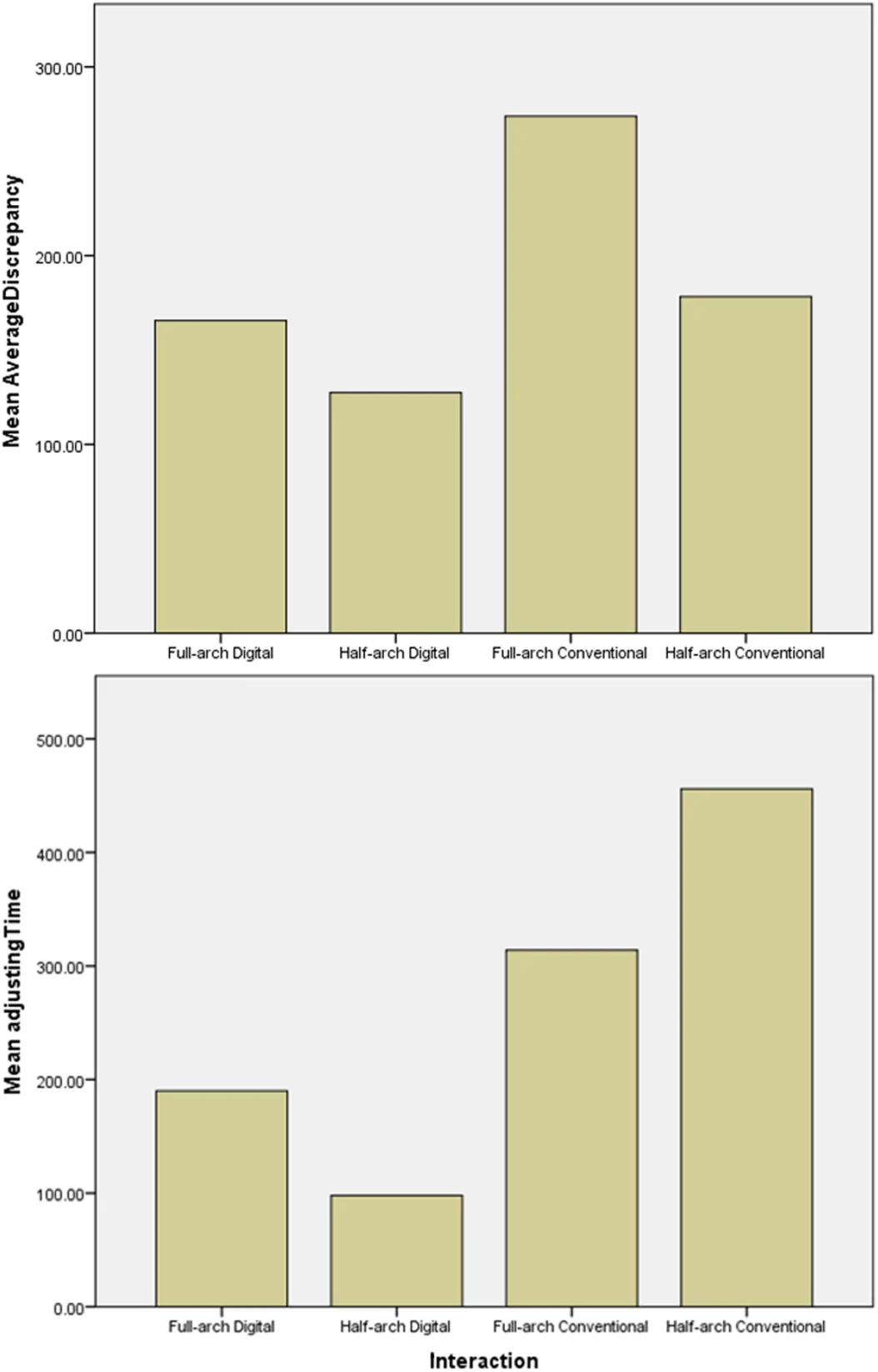
Adjustment amount (µm) and time (s) in all groups.

Post‐hoc comparisons (Table 1) showed that CF required significantly more occlusal adjustment than DH (*p* = 0.026) but significantly less chairside adjustment time than CH (*p* = 0.004); both CF and CH took longer to adjust than DF and DH (all *p* < 0.05). These trends are depicted in Figure 6.

**Table 1:** Pair‐wise comparisons between different groups.

Group A	Group B	N (each group)	Adjustment amount (µm)			Adjustment time (s)		
			Means (a–b)	Mean Δ ± SD	*p* value	Means (a–b)	Mean Δ ± SD	*p* value
DF	DH	5	165.56–127.42	+38.14 ± 36.38	0.837	190–198	+92.00 ± 32.92	0.073
	CF	5	165.56–273.88	−108.32 ± 54.80	0.124	190–314	−124.00 ± 32.65	0.012*
	CH	5	165.56–178.26	−12.70 ± 30.78	0.992	190–456	−266.00 ± 21.12	< 0.001*
DH	CF	5	127.42–273.88	−146.46 ± 56.82	0.026*	98–314	−216.00 ± 44.16	< 0.001*
	CH	5	127.42–178.26	−50.84 ± 34.24	0.687	98–456	−358.00 ± 36.47	< 0.001*
CF	CH	5	273.88–178.26	+95.62 ± 53.41	0.198	314–456	−142.00 ± 36.22	0.004*

Abbreviations: CF, conventional full‐arch; CH, conventional half‐arch; DF, digital full‐arch; DH, digital half‐arch; “Mean Δ”, mean difference Group A–Group B; SD, standard deviation.

* Statistically significant at *α* = 0.05.

### 3.2 Aggregated Analyses

According to Table 2, Independent‐samples *t*‐tests confirmed that the digital workflow required significantly less occlusal adjustment (*p* = 0.035) and chairside time (*p* < 0.001) than the conventional workflow. In contrast, arch span (full vs. half) did not significantly influence either outcome.

**Table 2:** Combined comparisons between different methods.

Group A	Group B	*N* (each group)	Adjustment amount (µm)			Adjustment time (s)		
			Means (a–b)	Mean Δ ± SD	*p* value	Means (a–b)	Mean Δ ± SD	*p* value
Digital (combined)	Conventional (combined)	10	146.49–226.07	−79.58 ± 34.96	0.035*	144–385	−241 ± 36.43	< 0.001*
Full‐arch (combined)	Half‐arch (combined)	10	219.72–152.84	66.88 ± 36.41	0.083	252–277	−25 ± 67.22	0.71

Abbreviations: “Mean Δ”, mean difference Group A–Group B; SD, standard deviation.

* Statistically significant at *α* = 0.05.

## 4 Discussion

The results of the present study demonstrated a significant difference between digital and conventional impression groups in terms of the average discrepancy parameter, with less occlusal adjustment required in the digital group. Occlusal adjustment time was also significantly lower in the digital group. The amount of occlusal adjustment and the adjustment time between full‐arch and half‐arch groups showed no significant difference.

Among all groups, DH ideally required both the least occlusal adjustment amount and time, CF required the greatest occlusal adjustment amount, and CH required the longest adjustment time. In pairwise comparisons, only the DH group and CF group showed a significant difference in terms of average discrepancy. For adjustment time, significant differences existed between all groups, except for DH and DF groups comparison.

Several studies report higher accuracy with digital methods compared to conventional impressions (Joda and Brägger 2015; Joda et al. 2016; Maestre‐Ferrín et al. 2012). Joda et al. (2016) showed that digital methods required significantly fewer clinical adjustments and shorter adjustment times, consistent with our findings. They confirmed superior clinical accuracy of digital methods specifically for cement‐retained implant restorations, cautioning against generalizing results to all restoration types. Chen et al. (2025) recently demonstrated that intra‐oral scanning provides occlusal contacts equal to or better than closed‐tray impressions for single posterior implant crowns. Existing evidence is largely restricted to single‐unit cases, underscoring the need to validate these findings in multi‐unit or splinted restorations such as ours.

The magnitude of time savings depends on workflow details. In monolithic zirconia screw‐retained crowns, Derksen et al. (2021) reported faster adjustments with digital scans, yet cautioned that total chairside time can shift when full‐versus half‐arch impressions, glazing, staining, or even the clinician's willingness to refine contacts are factored in. Such variables help explain why digital benefits are more pronounced in our controlled comparison of two‐unit splinted restorations.

Digital contacts are not only faster to seat but closer to the intended design. Ren et al. (2021) compared occlusal and proximal contact accuracy of implant crowns fabricated using four protocols. They used digital scans with short and long PEEK scan bodies and conventional impressions (open‐ and closed‐tray techniques). They found no significant differences between digital scan‐body groups, but digital methods generally achieved closer‐to‐ideal contacts, similar to our occlusal findings. Lower occlusal surface adjustments in digital protocols preserved occlusal morphology, leading to contact distribution closer to the original design. Greater occlusal adjustments can alter contacts significantly, necessitating remakes, additional patient visits, reduced patient satisfaction, potential microcracks, and compromised restoration strength. Furthermore, repeated firings can affect the color and strength of restorations. Gjelvold et al. (2016), comparing digital and conventional impressions for fixed dental prostheses, reported better occlusal contacts and reduced adjustments in digital groups. Conventional inaccuracies, such as altered closure paths due to registration materials, dimensional instability, and mounting errors, explained these differences, aligning with our study outcomes. Although digital workflows potentially increase overall laboratory processing time, their simplicity, reduced clinical adjustments, saved chairside time, and lowered risk of porcelain cracks and chipping are considerable advantages (Joda et al. 2016).

Building on earlier evidence, Biadsee et al. (2025) demonstrated near‐perfect concordance between virtually planned and clinically verified contacts, confirming CAD accuracy intra‐orally. Peng et al. (2025) reported that digitally fabricated occlusal devices required the smallest volumetric adjustments at delivery, translating directly into time savings. Extending these benefits to occlusal splints, Blasi et al. (2023) showed CAD‐CAM splints needed markedly fewer post‐delivery modifications than analog counterparts, again underscoring superior contact fidelity. Collectively, these convergent findings strengthen our observation that reduced grinding is an inherent advantage of digital workflows.

While most reports agree that fully digital protocols shorten overall treatment time, yet their impact on occlusal‐adjustment time is inconsistent. The study by Pan et al. (2019) measured total clinical time (including impression and crown delivery) and showed digital workflows were overall faster, but, unlike our study, the difference was not significant. The likely cause is methodological: their single‐unit, screw‐retained zirconia crowns were placed in non‐free‐end sites with full‐arch scans and mixed implant levels, whereas we evaluated two‐unit splinted PMMA crowns in a mandibular free‐end site and compared both half‐ and full‐arch scans under bone‐level conditions. Echoing our posterior free‐end findings, a self‐controlled study on implant‐supported single crowns reported that CAD‐designed restorations achieved occlusal contacts and clearances at least as accurate as conventional wax‐ups (He et al. 2023).

On the other hand, several trials have failed to detect a digital advantage in occlusal refinement. Zhang et al. (2021) compared the occlusal surfaces of zirconia crowns fabricated using digital and conventional methods for 12 patients and reported no significant differences between groups regarding adjustment time, occlusal contact distribution, or patient satisfaction. They added dynamic occlusal adjustments to the CAD workflow, effectively equalizing chairside work. Hashemi et al. (2022) found no significant difference between digital and conventional methods regarding occlusal contacts and adjustment times in implant‐supported crowns. They judged discrepancies ≤ 350 µm clinically acceptable, a threshold above the mean adjustments recorded in all of our groups. Such protocol choices, together with differences in scanner models, operator experience, and crown materials, can mask benefits that emerge under stricter acceptance criteria. Besides, clinical adjustment in patients might also differ from adjustments on laboratory models, potentially influencing outcomes. Additionally, variability in virtual‐articulator software accuracy and usability, as shown by a recent systematic review, offers another plausible explanation for discrepancies across studies (Saini et al. 2025).

The occlusal adjustment needs reported in our study differ from certain other studies that found CAD‐CAM monolithic crowns required no occlusal adjustments at all. This discrepancy may stem from various factors, including differences in implant type, scanner model, CAD‐CAM equipment, technician skill, and restoration type (Joda and Brägger 2015; Abduo et al. 2010; Joda, Ferrari, Brägger 2017; Joda, Ferrari, Gallucci, et al. 2017).

Yokosuka et al. (2021) evaluated implant‐supported crowns fabricated by digital and conventional methods. They compared implant superstructures for distance and angulation and measured articulator incisal pin displacement using a leaf gauge. They found no significant difference in abutment distance or angle between methods. However, digital methods had significantly greater incisal pin displacement than conventional, contrasting with our findings. This difference might arise because, in their study, crowns underwent preliminary adjustments on stone casts before placement on master models, unlike our protocol. Furthermore, only vertical displacement of the pin was considered, neglecting potential horizontal discrepancies.

Our study also compared full‐arch and half‐arch impression methods, finding no significant difference in adjustment times. However, including the impression‐taking duration might alter these findings. Overall, digital workflows can effectively reduce clinical adjustment time and occlusal adjustment amounts for implant‐supported restorations while preserving restoration accuracy and quality.

A recent systematic review and meta‐analysis reported a non‐significant trend toward shorter total clinical time with digital scans for complete‐coverage, fixed tooth‐supported prostheses, despite significantly greater patient comfort with digital methods (Bandiaky et al. 2022). Some recent studies even amplified the advantage of digital workflow by refining new methodologies. Ren et al. (2024) showed that incorporating an auxiliary occlusal device into a fully digital workflow for two‐implant crowns significantly reduced both occlusal deviation and laboratory time compared with conventional workflow.

This in‐vitro study used acrylic models, a single scanner, one impression material, one bone‐level implant system, and PMMA crowns only; hence its findings may not fully translate to diverse clinical scenarios. Future in‐vivo trials should assess free‐end restorations with multiple implant systems, splinted frameworks, various restorative materials, and include T‐Scan‐based contact analyses as well as impression‐taking time to provide a more comprehensive evaluation.

## 5 Conclusion

Within the limits of this in‐vitro study, digital impressions required significantly less occlusal adjustment and chairside time than conventional impressions. Adjustment amount and time showed no significant differences between full‐ and half‐arch scans; however, within conventional workflows the full‐arch was faster than the half‐arch, with comparable adjustment amounts. Among all protocols, the digital half‐arch demanded the least adjustment and the shortest time. Collectively, these results indicate that digital—particularly digital half‐arch—workflows can trim chairside procedures, better preserve occlusal morphology, and heighten patient and clinician satisfaction, thereby guiding clinicians toward more efficient splinted‐implant prosthetic workflows.

## Author Contributions

Mohammadreza Nakhaei and Hossein Dashti conceptualized and designed the study. Mahsa Rahmani and Arsalan Shahri performed the experimental procedures. Arsalan Shahri and Ali Kazemian conducted the statistical analysis. Saeid Sabzevari and Masih Rezaee drafted the manuscript, and all authors revised and approved the final version.

## Ethics Statement

The protocol of this study was approved by the Research and Ethics Committee of Mashhad University of Medical Sciences (IR.MUMS.DENTISTRY.REC.1401.023).

## Conflicts of Interest

The authors declare no conflicts of interest.

## Data Availability

The data that support the findings of this study are available from the corresponding author upon reasonable request.
